# *Giardia* spp. in Brazil at human–animal–environment interfaces: a systematic review from a One Health perspective

**DOI:** 10.3389/fpara.2026.1895842

**Published:** 2026-08-10

**Authors:** Mirella Andrade Fonseca, Elaine Santana Gonçalves Sarti, Elizângela Guedes

**Affiliations:** 1 Independent Researcher, Três Pontas, Minas Gerais, Brazil; 2 Independent Researcher, Caconde, São Paulo, Brazil; 3 Department of Veterinary Medicine, Universidade Professor Edson Antônio Velano (UNIFENAS), Alfenas, Minas Gerais, Brazil

**Keywords:** assemblages, epidemiology, *Giardia duodenalis*, giardiasis, neglected tropical diseases, sanitation, zoonoses

## Abstract

**Introduction:**

Giardiasis, caused by *Giardia duodenalis*, remains one of the most relevant enteric parasitic diseases in Brazil, affecting both humans and animals. This systematic review aimed to analyze the occurrence of *Giardia* spp. in Brazil from a One Health perspective, integrating epidemiological and molecular evidence related to parasite circulation among different hosts and environments.

**Methods:**

The review was conducted in accordance with the PRISMA 2020 guidelines, using searches in the PubMed, LILACS, and SciELO databases for studies published between 2016 and March 2026.

**Results:**

A total of 114 studies involving humans, animals, and environmental samples were included. Only nine of these studies (7.9%) adopted a genuinely integrated One Health design that simultaneously evaluated two or more compartments, and assemblage-level molecular characterization was available for only 17 studies (14.9%); these proportions should be considered when interpreting the One Health framing and the molecular inferences of this review. Substantial methodological heterogeneity was observed across studies, encompassing different coproparasitological, immunological, and molecular methods. In humans, occurrence was higher in vulnerable populations and in areas with inadequate basic sanitation. Among animals, dogs, cats, cattle, and wildlife stood out as important hosts. In environmental samples, a high frequency of detection was recorded in water, sewage, and food. Molecularly, assemblages A and B predominated, both associated with zoonotic and anthroponotic transmission.

**Discussion:**

The findings reinforce the importance of integrated surveillance and One Health approaches for the control of giardiasis in Brazil.

## 1 Introduction

Giardiasis, caused by *Giardia duodenalis* (syn. *G. lamblia* and *G. intestinalis*), is among the most frequent enteric parasitic diseases worldwide and is considered a major public health concern, particularly in regions with inadequate sanitary conditions and greater socioeconomic vulnerability. It affects both humans and various animal groups, with clinical manifestations ranging from asymptomatic cases to persistent diarrhea, intestinal malabsorption, and impaired child development (Beltrão et al., 2022; Adam, 2021).

Transmission occurs predominantly through the fecal–oral route via contaminated water, food, fomites, and direct person-to-person or animal-to-human contact. However, the epidemiology of giardiasis is considerably more complex than a simple waterborne disease, as domestic animals, wildlife, and environmental reservoirs may all contribute to the maintenance and dissemination of the parasite. This interconnected transmission network reinforces the importance of considering the human–animal–environment interface when investigating the epidemiology and control of *Giardia* infections (Wang et al., 2023).

From a molecular perspective, *G. duodenalis* exhibits considerable genetic diversity and is currently classified into eight assemblages (A–H), which differ in host range and zoonotic potential. Assemblages A and B are the most frequently reported in humans but have also been identified in several animal species, whereas the remaining assemblages are generally associated with specific host groups, although occasional cross-species infections have been documented (Davoodi et al., 2025). Molecular characterization therefore provides valuable information for understanding transmission pathways, identifying potential reservoirs, and assessing the epidemiological significance of different host species.

Within this context, the One Health approach provides an appropriate framework for studying giardiasis because it recognizes the close interdependence between human, animal, and environmental health. By integrating these components, One Health facilitates a more comprehensive understanding of parasite transmission dynamics, zoonotic interfaces, and environmental contamination, thereby supporting more effective surveillance and control strategies (Wang et al., 2023; Davoodi et al., 2025).

Brazil represents a particularly relevant setting for investigating giardiasis because of its remarkable environmental, climatic, ecological, and socioeconomic heterogeneity. The country encompasses six major biomes, one of the world’s greatest biodiversities, extensive livestock production systems, rapid urbanization, marked regional inequalities in sanitation coverage, and populations experiencing different levels of social vulnerability, including Indigenous, Quilombola, riverine, and rural communities (Köster et al., 2021; Zenazokenae et al., 2019; Eustachio et al., 2019; Fantinatti et al., 2016, 2023) Together, these characteristics create diverse epidemiological scenarios that may influence the transmission dynamics, persistence, and distribution of *Giardia* spp. across different regions of the country.

Although previous reviews have addressed important aspects of giardiasis in Brazil, particularly the review by Fantinatti et al. (2020), the growing number of studies published during the last decade has substantially expanded the available evidence regarding molecular epidemiology, environmental contamination, animal reservoirs, and zoonotic transmission.

Nevertheless, the Brazilian literature remains largely compartmentalized, with most investigations focusing exclusively on humans, animals, or environmental matrices. Consequently, studies simultaneously evaluating these three interconnected components under a One Health framework remain scarce, limiting a comprehensive understanding of transmission pathways and epidemiological connectivity across different hosts and environmental reservoirs. A comprehensive and updated synthesis of the available evidence is therefore needed to identify current knowledge gaps, support integrated surveillance strategies, and provide a more complete understanding of the epidemiology of *Giardia* spp. in Brazil (Eustachio et al., 2019; Fantinatti et al., 2020; Köster et al., 2021; Harvey et al., 2020).

Accordingly, this systematic review aimed to synthesize and critically analyze the available evidence on the occurrence, geographical distribution, and molecular diversity of *Giardia* spp. in humans, animals, and environmental matrices in Brazil from a One Health perspective. Furthermore, the review sought to identify the *G. duodenalis* assemblages reported in the country, discuss their zoonotic implications and epidemiological significance, identify current knowledge gaps, and provide evidence to support future surveillance, prevention, and research strategies.

## 2 Methodology

This systematic review was conducted in accordance with the recommendations of the PRISMA 2020 guidelines (Preferred Reporting Items for Systematic Reviews and Meta-Analyses), with the aim of compiling and analyzing scientific evidence on the occurrence and distribution of *Giardia* spp. in Brazil from a One Health perspective. The review protocol was not prospectively registered. The research question was formulated as follows: “What is the occurrence and distribution of *Giardia* spp. in humans, animals, and environmental samples in Brazil, from a One Health perspective?”

Original peer-reviewed observational studies investigating the occurrence of *Giardia* spp. or *Giardia duodenalis* in humans, animals, or environmental samples in the Brazilian context were eligible for inclusion. Observational studies with cross-sectional, cohort, case–control, and environmental monitoring designs were considered eligible. Given the predominantly cross-sectional design of the included studies, no minimum follow-up duration was established as a criterion. Review articles, case reports, exclusively laboratory-based experimental studies, and publications that did not present relevant epidemiological data on *Giardia* spp. as a primary or secondary variable of interest were excluded. No language restriction was applied.

The literature search was conducted in the PubMed (MEDLINE interface), LILACS (accessed through the Virtual Health Library — VHL), and SciELO databases with the last search conducted on March 21, 2026. Study registries, data repositories, reference lists of included studies, and forward and backward citation searching were not consulted. Authors and experts were not contacted for the identification of additional studies. The search strategy was structured using the descriptors *Giardia*, *Giardia duodenalis*, giardiasis, prevalence, and Brazil, combined through the Boolean operators AND and OR, adapted to the syntax of each database. The following search strategies were applied: PubMed: (“Giardia” OR “giardiasis”) AND (“Brazil” OR “Brasil”); LILACS: (giardíase OR Giardia) AND (Brasil OR Brazil); and SciELO: Giardia AND Brazil. Studies published from January 2016 onwards were considered eligible to capture recent epidemiological evidence as well as advances in diagnostic and molecular approaches to giardiasis in Brazil. Grey literature, theses and dissertation repositories, and forward and backward citation searching were not included; the review was deliberately restricted to peer-reviewed studies indexed in the selected databases to ensure methodological consistency and verifiable sourcing, which is acknowledged as a potential limitation regarding the coverage of non-indexed Brazilian literature.

**Figure 1:**
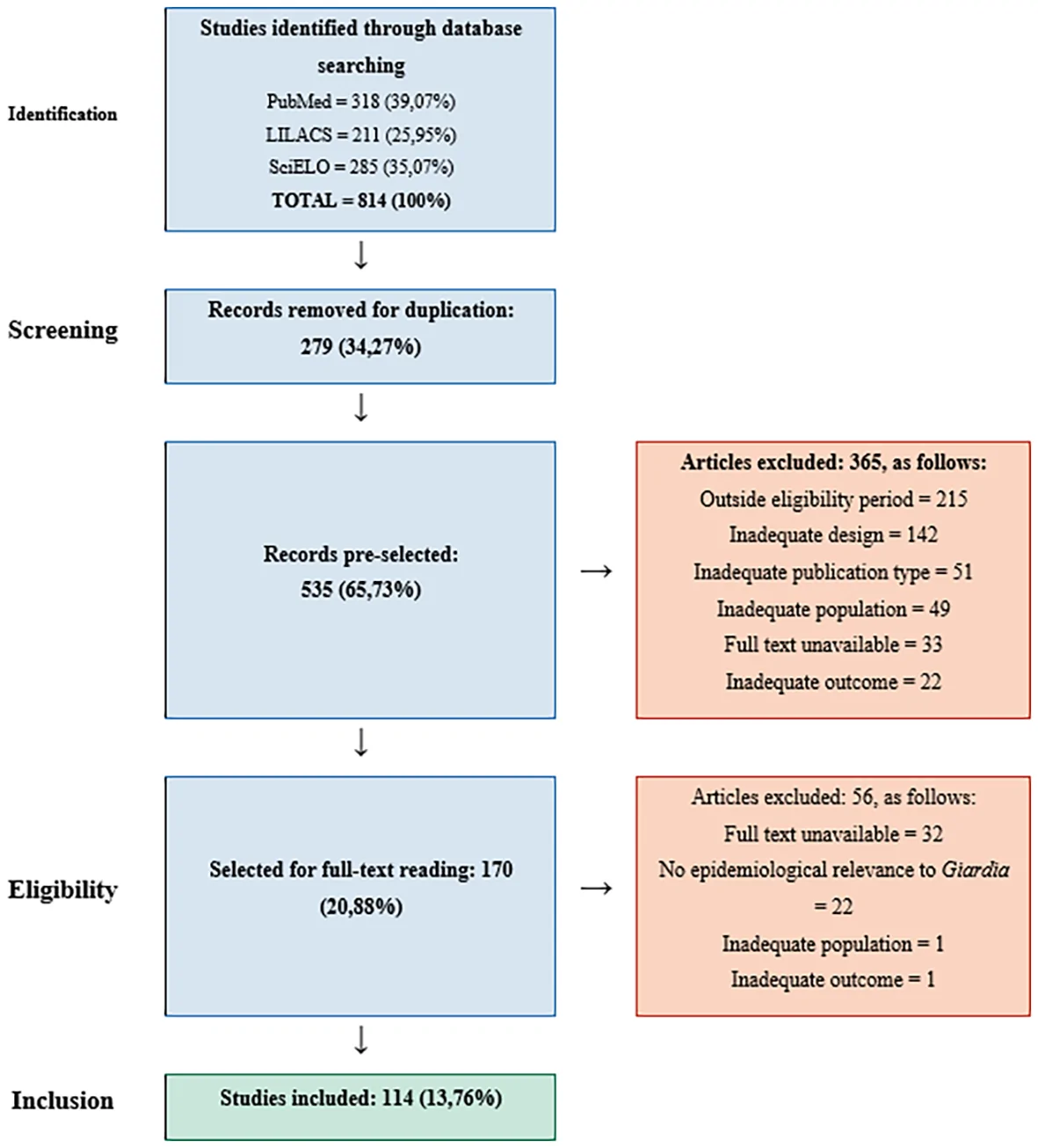
PRISMA 2020 flow diagram of the study selection process.

The study selection process followed the PRISMA 2020 recommendations and is summarized in **Figure 1**.

Study selection and data extraction were performed by the principal author and subsequently verified by the co-authors, using a standardized data extraction form; independent double screening was not applied. The following information was extracted from each study: first author, year of publication, study location, population or environmental matrix investigated, sample size, diagnostic method, occurrence or prevalence of *Giardia* spp., and *G. duodenalis* assemblages when molecular characterization was available.

A formal assessment of methodological quality or risk of bias was not performed because the primary objective of this review was to provide a comprehensive descriptive synthesis of the occurrence of *Giardia* spp. across heterogeneous human, animal, and environmental studies rather than to estimate pooled effect sizes. This should be considered when interpreting the findings.

The included studies were synthesized using a narrative and descriptive approach, organized into three categories according to the population investigated: human, animal, and environmental. Individual study data were compiled into a structured table presented in the main text, summarizing the principal extracted variables. Because of the marked clinical, methodological, and epidemiological heterogeneity among the included studies—including differences in study populations, diagnostic methods, sample types, and study designs—quantitative synthesis (meta-analysis) was considered inappropriate. Heterogeneity was explored qualitatively by comparing study populations, geographic regions, diagnostic methods, sample types, and reported outcomes across the included studies.

## 3 Results and discussion

### 3.1 Search strategy and characterization of the included studies

The literature search identified 814 records across the PubMed (n = 318), LILACS (n = 211), and SciELO (n = 285) databases. After duplicate removal and eligibility assessment according to the predefined inclusion criteria, 114 studies were included in the qualitative synthesis. The complete process of identification, screening, eligibility, and inclusion is presented in the PRISMA 2020 flow diagram (**Figure 1**).

The included studies were categorized according to the epidemiological compartment investigated: human-only studies (n = 39), animal-only studies (n = 43), environmental studies (n = 23), and integrated One Health studies (n = 9), the latter comprising investigations that simultaneously evaluated two or more compartments (human, animal, and/or environmental interfaces). Their methodological characteristics are summarized in **Table 1**.

**Table 1:** Methodological characterization and categorization of the studies included in the systematic review on *Giardia* spp. in Brazil (2016–2026).

Variable	Characterization
Publication period	2016–2026
Total of included studies	114
Databases consulted	PubMed, LILACS e SciELO
Screening and management tool	*Rayyan*
Type of study	Systematic literature review
Methodological guideline	PRISMA 2020
Study designs included	Cross-sectional, retrospective, cohort, and case–control observational studies, and environmental studies.
Exclusion criteria	Reviews, case reports, exclusively experimental laboratory studies, and articles without relevant epidemiological data.
Parasitological methods	Conventional optical microscopy, coproparasitological concentration and sedimentation techniques.
Immunological methods	ELISA, direct immunofluorescence, and immunochromatographic tests.
Molecular methods	Conventional PCR, nested PCR, real-time PCR, and genetic sequencing.
Extracted variables	Author, year, location, population, sample type, diagnostic method, prevalence/frequency, and assemblages.
Studies in humans	39
Studies in animals	43
Environmental studies	23
Studies with One Health interface (human–animal–environment)	09*
Analytical strategy	Qualitative narrative synthesis.
Meta-analysis	Not performed due to methodological heterogeneity.

PCR, Polymerase Chain Reaction; ELISA, Enzyme-Linked Immunosorbent Assay. *Includes studies with integrated assessment among humans, animals, and the environment, or with explicit epidemiological interface.

Source: Authors.

Overall, the evidence base was characterized by a predominance of cross-sectional study designs and marked methodological heterogeneity. Diagnostic approaches varied widely, including parasitological, immunological, and molecular techniques applied either independently or in combination. This methodological diversity, together with differences in study design, sampling strategies, and population characteristics, precluded quantitative synthesis through meta-analysis and supported the adoption of a qualitative narrative approach. Pooling within more homogeneous subgroups, for example studies applying the same diagnostic method within the same host category, was considered; however, the small number and uneven distribution of comparable studies within such subgroups, together with differences in sampling frames and reporting formats, did not allow a reliable subgroup meta-analysis.

Part of the variability observed among studies may also reflect differences in the diagnostic performance of the methods employed. Conventional microscopy generally presents lower sensitivity, particularly in infections with intermittent cyst shedding or low parasite burdens, whereas immunological assays and molecular techniques tend to provide higher diagnostic sensitivity, although their availability and application remain heterogeneous across Brazil. Consequently, differences in diagnostic methodology probably contributed to the variability in reported occurrence estimates observed among the included studies (Garcia and Shimizu, 1997; Ryan and Cacciò, 2013). This pattern is corroborated at the continental scale: in a meta-analysis of giardiasis in African children, the pooled prevalence estimated by real-time PCR (36.6%) markedly exceeded that obtained by microscopy (16.3%) (Kalavani et al., 2024), reinforcing that the diagnostic approach strongly shapes reported prevalence.

Beyond methodological heterogeneity, the distribution of the included studies demonstrates that giardiasis research in Brazil remains largely compartmentalized. Although studies were identified in human, animal, and environmental populations, only nine investigations simultaneously evaluated two or more of these epidemiological compartments. Consequently, most of the available evidence describes isolated transmission scenarios, limiting a comprehensive understanding of the interactions between humans, animals, and the environment under a One Health framework. This finding represents one of the principal knowledge gaps identified in the present review.

### 3.2 Occurrence of *Giardia* spp. in humans

The studies conducted in human populations demonstrated a broad distribution of *Giardia* spp. throughout the Brazilian territory, with records in all regions of the country and substantial heterogeneity regarding observed prevalence, diagnostic methods, and populations investigated. The regional synthesis of human studies is presented in **Table 2**.

**Table 2:** Regional synthesis of the occurrence of *Giardia* spp. in humans in Brazil according to studies included in the systematic review (2016–2026).

Region	Reported prevalence (%)	Predominant methods	Main epidemiological findings	Included studies
North	26,0%	Microscopy	High occurrence in a low-income community in the Brazilian Amazon, with *Giardia* identified as the most prevalent parasite.	Cardoso et al. (2017)
Northeast	1,21% – 35,5%	Microscopy, ELISA, PCR	Frequent occurrence in children, rural communities, peri-urban communities, incarcerated populations, and occupationally exposed populations, associated with socioeconomic and sanitary vulnerability.	Coronato-Nunes et al. (2017); Crisostomo et al. (2019); Dos Santos Lima et al. (2024); Dias et al. (2018); Ibiapina et al. (2020); Lima et al. (2019); Alves et al. (2021); Monteiro et al. (2018); Oliveira et al. (2020); Passos Neto et al. (2025); Santos Junior et al. (2023); Santos et al. (2020); Vargas et al. (2023)
Central-West	3,9% – 21,7%	Microscopy, PCR, sequencing	Occurrence recorded in Indigenous populations, urban communities, incarcerated individuals, solid waste pickers, and patients with comorbidities, with emphasis on the Tapirapé Indigenous population.	Curval et al. (2017); Higa Júnior et al. (2017); Köster et al. (2021); Machado et al. (2018); Rodrigues et al. (2019); Zenazokenae et al. (2019)
Southeast	1,2% – 51,4%	Microscopy, immunofluorescence, PCR	Occurrence recorded in daycare centers, schoolchildren, vulnerable urban communities, collective institutions, food handlers, pregnant women, and children, with high prevalences in daycare centers in Rio de Janeiro and São Paulo.	Albuquerque and Souza (2017); Barbosa et al. (2018); Brauer et al. (2017); Calegar et al. (2020); Corrêa et al. (2020); Costa M, et al. (2018); Domingues et al. (2025); Fantinatti et al. (2016, 2020, 2023); Faria et al. (2017); Fonseca et al. (2017); Gondim et al. (2019); Iasbik et al. (2018); Ignacio et al. (2017); Martins P, et al. (2019)
South	1,44% – 13,1%	Microscopy, PCR	Occurrence observed in population-based studies in clinical laboratories, urban communities, border communities, and SUS users, with association to poor sanitation.	Ferlito and Dalzochio (2020); Ferreira et al. (2021); Motta et al. (2020); Seguí et al. (2018)

Values represent the range of prevalence observed among the studies included in each region.

Source: Authors.

The analysis of the regional distribution of the included studies reveals broad and heterogeneous occurrence of *Giardia* spp. in human populations in Brazil, with substantial variation in reported prevalences according to region, population characteristics investigated, and diagnostic methods employed.

In the North Region, a prevalence of 26.0% was identified in a low-income community in the Brazilian Amazon, associated with precarious socio-environmental conditions and limited basic sanitation (Cardoso et al., 2017).

In the Northeast Region, prevalences ranged from 1.21% to 35.5%, with higher frequency in children, rural communities, and peri-urban populations with limited sanitary infrastructure, reinforcing the relevance of fecal–oral transmission and exposure to contaminated water (Coronato-Nunes et al., 2017; Dos Santos Lima et al., 2024; Oliveira et al., 2020). Several studies reported high frequencies of *Giardia* spp. in pediatric populations and socially vulnerable communities. Alves et al. (2021) reported a prevalence of 27.6% among children and adolescents in Bahia, while Lima et al. (2019) found a prevalence of 21.2% among children with diarrhea in municipalities of the Brazilian semiarid region, reinforcing the association between giardiasis, inadequate sanitation, and socioeconomic vulnerability. Conversely, studies based on routine laboratory samples or general populations reported substantially lower prevalences (Crisostomo et al., 2019; Ibiapina et al., 2020; Passos Neto et al., 2025), suggesting that both population characteristics and study design contribute to the observed variability.

In the Central-West, although less represented quantitatively, prevalences between 3.9% and 21.7% were reported, with emphasis on Indigenous populations and scenarios of socio-environmental vulnerability. Köster et al. (2021) identified prevalences between 13.5% and 21.7% in Tapirapé Indigenous people in Mato Grosso, while Zenazokenae et al. (2019) observed a prevalence of 9.3% in the Haliti-Paresí Indigenous population, also in Mato Grosso, in a context of absent water treatment and poor sanitation. Machado et al. (2018) recorded a prevalence of 16% among diabetic individuals in the Federal District, evidencing relevant occurrence in populations with comorbidities.

In the Southeast and South Regions, prevalences ranged from 1.2% to 51.4% and from 1.44% to 13.1%, respectively, with marked occurrence in schoolchildren, daycare centers, collective institutions, and urban communities, suggesting persistence of interpersonal transmission and the influence of heterogeneous sanitary conditions. In a public daycare center in São Paulo, Corrêa et al. (2020) identified a prevalence of 51.4% in asymptomatic children, through the combination of microscopy and PCR. High frequencies were also observed by Fantinatti et al. (2016, 2023), with prevalences of 49.4% and 44.3% in children from urban communities in Rio de Janeiro. In contrast, laboratory-based and population-based studies reported lower prevalences: Costa M, et al. (2018), in an analysis of 6,289 samples in the metropolitan region of Belo Horizonte, identified an overall positivity of 1.2%, with higher occurrence among users of public laboratories (4.4%) compared with private laboratories (0.9%); and Iasbik et al. (2018), in the Zona da Mata region of Minas Gerais, recorded prevalences of 1.91% in Viçosa and 5.29% in Muriaé, in association with unfavorable sanitary and socioeconomic conditions.

A high frequency of asymptomatic infections was also observed, especially in children and collective settings, as reported by Corrêa et al. (2020); Calegar et al. (2020), and Fantinatti et al. (2020), which reinforces the potential for silent maintenance of transmission and hinders strategies based exclusively on clinical diagnosis.

The wide variation in reported occurrence across studies was accompanied by considerable differences in the populations investigated, epidemiological settings, and diagnostic methods employed. In addition, several studies consistently identified socio-environmental determinants associated with *Giardia* spp. occurrence, including lack of treated water, consumption of unsafe water, inadequate waste disposal, contact with contaminated soil, household overcrowding, and low maternal education (Coronato-Nunes et al., 2017; Ferreira et al., 2021; Rodrigues et al., 2019). Collectively, these findings reinforce the close relationship between giardiasis and social vulnerability across different Brazilian regions.

Collectively, the human studies consistently demonstrate that giardiasis remains closely associated with socioeconomic vulnerability, particularly among children, rural communities, Indigenous peoples, and Quilombola populations. Similar epidemiological patterns have been described in other low- and middle-income countries, where inadequate sanitation, limited access to safe drinking water, and poor hygiene conditions remain the principal determinants of transmission (Adam, 2021; Wang et al., 2023). These findings reinforce that improvements in water supply, sanitation, and health education remain essential components of giardiasis control within a One Health framework. Comparable epidemiological patterns have been documented across Latin America and Africa. In an updated systematic review of molecular reports from six Latin American countries, Sarria-Guzmán et al. (2022) found that research remained concentrated in Brazil and Colombia and that the highest prevalences were recorded in Brazil, mirroring the regional concentration of evidence observed in the present review. In Africa, Walana et al. (2025) reported a pooled giardiasis prevalence of 31.9%, predominantly in children and in association with deficient water, sanitation, and hygiene conditions, reinforcing that the social and sanitary determinants of giardiasis are shared across low- and middle-income settings. Consistently, a systematic review and meta-analysis of 114 studies from 29 African countries estimated a pooled prevalence of 18.3% among children, ranging from 0.08% in Cameroon to 65.1% in Niger, with research strongly concentrated in Ethiopia and Egypt and the highest burdens among iron-deficient, HIV-infected, displaced, and handicapped children (Kalavani et al., 2024). This geographical concentration of research parallels the uneven distribution of studies observed across Brazilian regions.

Overall, the available evidence indicates that human giardiasis in Brazil cannot be interpreted solely as an individual parasitic infection but rather as a consequence of interconnected environmental, social, and sanitary determinants. The persistence of infection in socially vulnerable populations highlights the importance of integrated public health policies combining improvements in sanitation, environmental management, health education, and epidemiological surveillance. These findings reinforce the relevance of the One Health approach for reducing transmission and mitigating the disproportionate burden of giardiasis among vulnerable communities.

### 3.3 Occurrence of *Giardia* spp. in animals

A total of 43 (37.7%) studies involving different animal species were identified, including dogs, cats, cattle, small ruminants, pigs, equids, rabbits, non-human primates, bats, birds, and other wild animals. A predominance of studies in companion animals was observed, particularly dogs and cats, followed by cattle and other species of zootechnical and sanitary interest (**Table 3**).

**Table 3:** Synthesis of studies on the occurrence of *Giardia* spp. in animals in Brazil (2016–2026).

Animal category	No. of studies	Main species evaluated	Reported frequency/detection	Included studies
Dogs	15	*Canis lupus familiaris*	0,75% – 50,0%	Arruda et al. (2023); Bricarello et al. (2018); Bricarello et al. (2020); Kipper et al. (2018); Lima et al. (2021); Moraes et al. (2019); Mortari et al. (2024); Oliveira-Arbex et al. (2016, 2022); Osmari et al. (2021); Reginaldo et al. (2025); Souza C, et al. (2023); Souza J, et al. (2023); Trevisan et al. (2020); Ugalde et al. (2023)
Cats	9	*Felis catus*	2,02% – 24,8%	Bricarello et al. (2020); Ferraz et al. (2021); Lima et al. (2021); Moraes et al. (2019); Mósena et al. (2019); Silva et al. (2023); Souza et al. (2017); Souza J, et al. (2023); Torrico et al. (2020)
Cattle and buffaloes	5	Dairy cattle, buffaloes, and calves	2,0% – 26,75%	Aquino et al. (2019); Santos et al. (2024); Sevá et al. (2018); Vilela et al. (2017); Volpato et al. (2017)
Small ruminants	3	Sheep and goats	6,6% – 20,0%	Calegar et al. (2023); Millar et al. (2020); Moraes et al. (2019)
Pigs	1	*Sus scrofa domesticus*	21,2%	Calegar et al. (2023)
Equids	1	Draft equids	5,3%	Costa P, et al. (2018)
Rabbits	1	*Oryctolagus cuniculus*	40,0%	Baptista et al. (2023)
Wildlife	9	Non-human primates, bats, sloths, wild mammals, wild carnivores, aquatic mammals, and wild birds	2,94% – 59,0%	Borges et al. (2017); Fehlberg et al. (2021); Lignon et al. (2025); Lima et al. (2021); Müller et al. (2025); Pantoja et al. (2019); Pereira Gonçalves et al. (2024); Reis et al. (2023); Reis et al. (2024)
Zoo and captive fauna	5	Mammals, birds, and reptiles in captivity	0% – 23,0%	Barbosa et al. (2019); Chagas et al. (2019); Gallo et al. (2018); Gallo et al. (2020); Prazeres Júnior et al. (2024)

Values represent the range of prevalence observed among the studies included in each animal category. Studies that evaluated more than one animal category were counted in each corresponding row; therefore, the sum of categories exceeds the total number of studies in animals (n = 43).

Source: Authors.

The animal studies included in this review evidenced the broad distribution of *Giardia* spp. in Brazil, encompassing domestic, production, and wild animals, with substantial variation in reported prevalences according to the species investigated, the rearing environment, and the diagnostic methods employed.

Dogs constituted the most frequently investigated category, with prevalences ranging from 0.75% to 50.0%. The highest frequencies were observed in animals kept in collective environments, such as shelters and veterinary hospitals, suggesting a direct influence of high population density, sanitary management, and environmental contamination. Kipper et al. (2018) recorded a positivity of up to 50% in dogs attended at a veterinary hospital in Blumenau, Santa Catarina, using the SNAP *Giardia* immunological test. Similarly, Souza C, et al. (2023) identified a significant association between infection and dogs from shelters in Cuiabá, Mato Grosso (OR = 12.57; p < 0.001). In Niterói, Rio de Janeiro, Ugalde et al. (2023) observed greater occurrence in adult animals.

In cats, prevalences ranged from 2.02% to 24.8%, with marked occurrence in domiciled and sheltered animals. Moraes et al. (2019) identified a higher frequency in felines (24.8%) compared with dogs and small ruminants evaluated at the same veterinary hospital in Botucatu, São Paulo. Torrico et al. (2020) and Silva et al. (2023) reported prevalences of 17.96% and 12.2% in feline populations in Paraná and Rio de Janeiro, respectively These findings indicate that domestic cats may contribute to the maintenance of *Giardia* spp. in urban transmission cycles, particularly in environments characterized by close contact with humans and other companion animals.

Among production animals, cattle and buffaloes presented prevalences between 2.0% and 26.75%, especially on dairy farms and in intensive rearing systems. Factors such as young age, inadequate water management, intensive production systems, and environmental fecal contamination were frequently associated with infection, indicating that management practices may play an important role in parasite transmission within dairy production systems. Volpato et al. (2017), evaluating 43 dairy farms in Santa Catarina, identified a prevalence of 26.75% in calves, with an association with the manner of milk provision. Santos et al. (2024) also reported relevant frequencies in cattle and sheep in Rio Grande do Sul, reinforcing the circulation of the parasite in young herds.

Small ruminants presented prevalences between 6.6% and 20.0% (Moraes et al., 2019; Millar et al., 2020; Calegar et al., 2023). Equids and rabbits were evaluated in isolated studies, with prevalences of 5.3% and 40.0%, respectively (Costa P. et al., 2018; Baptista et al., 2023).

In wildlife, prevalences ranged from 2.94% to 59.0%, encompassing non-human primates, bats, sloths, aquatic mammals, wild carnivores, and birds. Pereira Gonçalves et al. (2024) identified a prevalence of 59.0% in non-human primates in southern Brazil, with greater frequency in animals kept in captivity (83.3%) than in free-living animals (20.0%), suggesting that confinement and increased interspecific contact may facilitate parasite transmission. Although most wildlife studies were descriptive, their findings demonstrate that free-ranging and captive wild animals may participate in the maintenance of *Giardia* spp. in different ecosystems, reinforcing the need for integrated surveillance at the wildlife–livestock–human interface.

In animals kept in zoos and captivity, prevalences ranged from 0% to 23.0% (Barbosa et al., 2019; Chagas et al., 2019; Gallo et al., 2018, 2020; Prazeres Júnior et al., 2024). These studies suggest that confinement, proximity between species, and continuous exposure to shared environments may facilitate parasite maintenance and environmental dissemination.

The wide distribution of *Giardia* spp. among companion animals, livestock, and wildlife highlights the ecological complexity of parasite circulation in Brazil. However, most studies evaluated animal populations independently, limiting a comprehensive assessment of transmission interfaces with humans and the environment. Consequently, although the frequent detection of the parasite in species living at the interface between human activities and natural ecosystems supports the potential contribution of animals to parasite maintenance, the relative epidemiological importance of each host group remains insufficiently characterized. These findings reinforce the need for integrated studies combining human, animal, environmental, and molecular data to better understand transmission pathways within a One Health framework.

### 3.4 Occurrence of *Giardia* spp. in environmental and food samples

Environmental studies accounted for 23 (20.2%) of the 114 included investigations and comprised analyses of raw and treated water, sewage, sludge, recreational waters, soil, sand, fresh produce, and other environmental matrices potentially involved in the transmission of *Giardia* spp. (**Table 4**).

**Table 4:** Main studies on the occurrence of *Giardia* spp. in environmental samples in Brazil included in the systematic review (2016–2026).

Environmental matrix	No. of studies	Localities	Reported frequency/detection	Included studies
Drinking water and supply systems	8	Goiás; Belo Horizonte-MG; metropolitan region of São Paulo; Vitória-ES; Canavieiras-BA; Foz do Iguaçu-PR; southern Brazil	33.3% – 100% in raw water; residual detection in treated water	Araújo et al. (2024); Keller et al. (2024); Lopes et al. (2017); Oliveira et al. (2024); Scherer et al. (2022); Silva and Scalize (2020); Silva et al. (2021); Visentini and Tagliari (2024)
Sewage, sludge, and biosolids	4	Londrina-PR; Ibiporã-PR; São Paulo	6.1% – 100% (raw sewage); 70% – 76% (treated sewage); 33.3% (biosolids)	Bonatti and Franco (2016); Ladeia et al. (2018); Ladeia et al. (2022); Martins et al. (2019)
Recreational environments (swimming pools and beach sand)	2	Campinas-SP; São Luís-MA	19.0% (swimming pools); 83.3% (beach sand)	Pineda et al. (2020); Viana et al. (2023)
Soil and sediments	2	Pelotas-RS; Caí and Paranhana rivers, RS	1.63% (soil); ~5% of parasitic forms detected (sediments)	Capella et al. (2018); Heck et al. (2021)
Food (vegetables and fruits)	4	Santo Antônio de Jesus-BA; Passo Fundo-RS; São Miguel do Oeste-SC; Londrina-PR	0% – 33,3%	Amor et al. (2023); Barancelli et al. (2025); Pinto-Ferreira et al. (2020); Vidigal and Landivar (2018)
Surfaces and fomites	2	Diamantina-MG	3.6% – 4.4% of biological forms detected	Andrade et al. (2017); Costa M, et al. (2018)
Public areas (environmental fecal samples)	1	Southwestern Goiás	3.2% (with positivity in 91.5% of the areas evaluated)	Martins et al. (2025)

Values represent the range of prevalence observed among the studies included in each environmental matrix.

Source: Authors.

The environmental studies included in this review evidenced the broad dissemination of *Giardia* spp. in different environmental matrices in Brazil, with substantial variation in detection frequencies according to sample type, locality, and diagnostic methods employed. The highest occurrences were recorded in water resources intended for human supply and in environments impacted by anthropogenic activities.

Among the studies related to drinking water and supply systems, a high frequency of detection was observed in surface water sources, public reservoirs, and treatment plants, with prevalences ranging from 33.3% to 100% in raw water. Silva and Scalize (2020) detected the parasite in 86.66% of raw water samples from surface sources in Goiás, with concentrations of up to 116.7 cysts/L and greater contamination in areas of agricultural and livestock activity. Araújo et al. (2024), in monitoring conducted in the metropolitan region of São Paulo, identified positivity in 100% of surface water samples and in 85.7% of reuse water samples, with molecular characterization of sub-assemblages AII, BIII, and BIV, indicating predominantly anthropogenic origin. Visentini and Tagliari (2024), through Quantitative Microbial Risk Assessment, estimated a maximum annual infection risk of 5.04 × 10^-^³ per person in raw water in southern Brazil, a value up to 50 times higher than the USEPA recommended limit.

The persistence of cysts after conventional treatment processes was consistently demonstrated. Keller et al. (2024) detected *Giardia* spp. in up to 100% of raw water samples and in water treated by filtration in Espírito Santo, with concentrations of up to 20.0 cysts/L in the treated effluent. Oliveira et al. (2024) observed positivity in 41.6% of raw water samples and in 16.6% of treated samples in the water supply system of Canavieiras, Bahia. Silva et al. (2021) identified average removal efficiency of only 1.27 log in treatment plants in Goiás, with risk modeling indicating an annual infection probability of 100%. This estimate derives from a single Quantitative Microbial Risk Assessment applied to one treatment system, and comparable risk estimates were not available for other water matrices or localities among the reviewed studies; it should therefore be interpreted as an illustrative, locality-specific finding rather than a generalizable risk value. Scherer et al. (2022) detected the parasite in raw, treated, and consumption water in Foz do Iguaçu, Paraná, including samples from pediatric environments.

In sewage, sludge, and biosolids matrices, the high frequency of detection reinforced the environmental persistence of the parasite. Ladeia et al. (2022) identified *Giardia* in 100% of raw sewage samples and in 70% of treated samples in Ibiporã, Paraná, with viable forms in 30% of the final effluent. Similar findings were reported by Martins F, et al. (2019), with positivity in 100% of raw sewage and 76% of treated sewage in Londrina, Paraná. Bonatti and Franco (2016) detected cysts in 33.3% of biosolid samples from treatment plants in São Paulo, with concentrations of up to 4,800 cysts/g, evidencing the potential of biosolids as environmental reservoirs. Ladeia et al. (2018) identified the parasite in filter backwash sludge samples from a treatment plant in Londrina, reinforcing its persistence in residues of the potabilization process.

In recreational environments, substantial occurrences were recorded in swimming pools and urban beach sand. Pineda et al. (2020) identified *Giardia* spp. in 19.0% of public swimming pools in Campinas, São Paulo, with molecular confirmation of assemblage A, associated with high user load and structural failures. Viana et al. (2023) detected *Giardia lamblia* in 83.3% of sand samples from urban beaches in São Luís, Maranhão, evidencing intense fecal contamination in collective-use environments.

In soil and sediment matrices, frequencies were lower but indicative of diffuse environmental contamination. Capella et al. (2018) detected *Giardia* spp. in 1.63% of soil samples from a vulnerable community in Pelotas, Rio Grande do Sul, in a context of widespread parasitic positivity. Heck et al. (2021) identified cysts in sediments from the banks of the Caí and Paranhana rivers, in Rio Grande do Sul, with greater contamination in urbanized areas, associated with inadequate basic sanitation.

In food, frequencies ranged from 0% to 33.3%, with greater occurrence in vegetables and fruits commercialized under inadequate hygienic-sanitary conditions. Amor et al. (2023) detected *Giardia* spp. in approximately 33.3% of strawberry samples commercialized in Santo Antônio de Jesus, Bahia. Vidigal and Landivar (2018) observed presence of the parasite in 23.7% of positive lettuce samples in self-service restaurants in São Miguel do Oeste, Santa Catarina. Barancelli et al. (2025) identified contamination in 1.5% of lettuce samples in Passo Fundo, Rio Grande do Sul. Pinto-Ferreira et al. (2020), in lettuce from conventional, organic, and hydroponic cultivation in Londrina, did not identify positivity for *Giardia* spp.

In surfaces and fomites, Costa M, et al. (2018) and Andrade et al. (2017) detected the parasite on banknotes and internal surfaces of buses in Diamantina, Minas Gerais, evidencing broad fecal contamination of everyday objects. Martins et al. (2025), in environmental fecal samples from public areas of 18 municipalities in southwestern Goiás, identified *Giardia* spp. in 3.2% of samples, with positivity in 91.5% of the areas evaluated, suggesting dispersed contamination and significant association with municipalities of smaller population size.

The recurrent detection of *Giardia* spp. in water, sewage, food, recreational environments, soil, and other environmental matrices demonstrates widespread fecal contamination and highlights the central role of the environment in sustaining parasite transmission. These findings are consistent with international evidence identifying contaminated water as one of the principal routes of exposure for both humans and animals (Wang et al., 2023). Furthermore, the persistence of the parasite after conventional water and sewage treatment processes, together with its detection in food, surfaces, and recreational environments, illustrates the complexity of transmission pathways in Brazil. Although molecular evidence remains limited, the identification of overlapping assemblages among human, animal, and environmental samples in some studies supports the existence of interconnected transmission interfaces. Nevertheless, most environmental investigations evaluated individual compartments in isolation, with few integrating environmental findings with concurrent human or animal epidemiological data. Furthermore, broader environmental drivers such as rapid urban expansion, land-use changes, inadequate sanitation infrastructure, and increasingly frequent extreme rainfall events may further influence environmental contamination and human exposure, although these factors were not directly evaluated in most of the included studies (Wang et al., 2023). Their incorporation into future One Health investigations may contribute to a more comprehensive understanding of giardiasis transmission in Brazil, reinforcing the need for integrated environmental surveillance.

### 3.5 Integrated studies from a One Health perspective

Although only nine studies adopted an integrated One Health approach, they provided important evidence regarding the concomitant circulation of *Giardia* spp. among humans, animals, and the environment in Brazil. The reported occurrence varied according to the epidemiological interface investigated, the socio-environmental context, and the diagnostic methods employed. (**Table 5**).

**Table 5:** Synthesis of integrated studies from a One Health perspective on the occurrence of *Giardia* spp. in Brazil (2016–2026).

Interface investigated	No. of studies	Localities	Main epidemiological findings	Included studies
Human–Animal–Environment	2	Ilhéus-BA; Paraná (dairy farms)	Simultaneous circulation of *Giardia* spp. in humans, animals, and environmental samples, with prevalences of 37.3% in children, 16.8% in dogs, and 5.2% in environmental samples (Harvey et al., 2020); detection in cattle (7.6%) and in drinking water, without positivity in humans (Toledo et al., 2017).	Harvey et al. (2020); Toledo et al. (2017)
Human–Animal	2	Ilhéus-BA; Teresina-PI	Identification of assemblages A, B, and E in children and predominance of assemblage E in dogs (Harvey et al., 2023); occurrence of *Giardia duodenalis* in humans (8.3%) with concurrent investigation in dogs, associated with poor sanitation (Silva et al., 2025).	Harvey et al. (2023); Silva et al. (2025)
Human–Environment	4	Rural Ceará; Sinop-MT; Diamantina-MG (Quilombola community); Cascavel-PR	Consistent association between occurrence in humans and environmental contamination, with human prevalences from 3.0% to 20.1%, contamination of recreational sand in 100% of samples, occurrence in water in Quilombola communities, and simultaneous detection in humans and water in the context of an epidemic outbreak involving 12,223 cases.	Carvalho et al. (2022); Dias et al. (2018); Eustachio et al. (2019); Marciano et al. (2022)
Animal–Environment	1	Santa Isabel-SP	Detection of *Giardia duodenalis* in 46.1% of bovine fecal samples and in up to 47.8% of water samples from shallow wells used for human consumption, evidencing animal–environmental cross-contamination.	Breternitz et al. (2024)

Integrated studies were considered those that investigated simultaneously two or more matrices (human, animal, or environmental) from a One Health perspective, with concomitant analysis of the interfaces evaluated.

Source: Authors.

The integrated studies from a One Health perspective, although representing only 9 (7.9%) of the included studies, provided important evidence regarding the concomitant circulation of *Giardia* spp. among humans, animals, and the environment in Brazil. Reported occurrence varied according to the epidemiological interface investigated, the socio-environmental context, and the diagnostic methods employed.

In studies that simultaneously evaluated the three matrices (human, animal, and environmental), Harvey et al. (2020) identified prevalences of 37.3% in children, 16.8% in dogs, and 5.2% in environmental samples in rural and semi-rural communities of Ilhéus, Bahia, evidencing broad circulation of the protozoan in direct interface between hosts and the environment. Toledo et al. (2017), in dairy farms in Paraná, recorded a prevalence of 7.6% in cattle, with greater occurrence in calves up to six months of age, and identified the protozoan in water samples used for consumption, frequently associated with the presence of fecal coliforms, although no cases were detected in human samples.

In the human–animal interface, Harvey et al. (2023), in continuity with the study conducted in Ilhéus, identified a giardiasis prevalence in children of up to 54.9% through the combination of parasitological and molecular methods, and up to 28.3% in dogs, with detection of assemblages A, B, and E in humans and predominance of assemblage E in canids, constituting the first report of this genotype in dogs in northeastern Brazil. Silva et al. (2025), in a rural community in Teresina, Piauí, identified *Giardia duodenalis* as the most frequent pathogenic protozoan in humans (8.3%), with concurrent investigation in 104 dogs, and a significant association between infection and contact with water bodies, inadequate waste disposal, and poor sanitation.

In the human–environment interface, a consistent association between the occurrence of the parasite in human populations and the contamination of environmental matrices was observed. Dias et al. (2018), in rural communities of Ceará, recorded prevalences in humans ranging from 3.0% to 20.1% over three years, with consumption of inadequate water in up to 70.9% of the households investigated. Carvalho et al. (2022) identified *Giardia lamblia* as the most prevalent parasite in children in Sinop, Mato Grosso, and detected positivity in 100% of sand samples from recreational areas, evidencing broad environmental contamination. Eustachio et al. (2019), in a Quilombola community in Diamantina, Minas Gerais, recorded *Giardia duodenalis* in 3% of individuals, in a context of universal contamination of water samples by fecal coliforms. Marciano et al. (2022), in an investigation of an acute diarrheal disease outbreak in Cascavel, Paraná, identified the protozoan in 6.4% of human fecal samples and in 3.8% of water samples, including backwash samples from the treatment plant, in an outbreak that involved 12,223 cases and was associated with the contamination of water sources by sewage.

In the animal–environment interface, Breternitz et al. (2024) investigated shallow wells in a rural area of Santa Isabel, São Paulo, identifying *Giardia duodenalis* in 26.0% of samples from a school well, 30.4% from a consumption tap, and 47.8% from a recreational area well, with concentrations of up to 32.85 cysts/L. In bovine feces collected simultaneously, positivity reached 46.1%, evidencing cross-circulation between herd and water sources used for human consumption.

Although integrated studies represented less than 10% of the investigations included in this review, they provided the strongest evidence of interactions among human, animal, and environmental compartments. Nevertheless, the limited number of studies simultaneously evaluating these interfaces demonstrates that giardiasis research in Brazil remains largely compartmentalized. This fragmentation restricts a comprehensive understanding of transmission pathways, particularly regarding the relative contribution of environmental contamination, domestic animals, livestock, and wildlife to parasite circulation. Future investigations integrating epidemiological, environmental, and molecular approaches will therefore be essential to clarify transmission dynamics and strengthen One Health surveillance strategies in Brazil.

### 3.6 Molecular diversity and distribution of *Giardia duodenalis* assemblages in Brazil

Molecular characterization of *Giardia duodenalis* isolates was performed in 17 of the 114 included studies (14.9%), encompassing human, animal, and environmental samples, with identification of assemblages A, B, C, D, and E, and of sub-assemblages AI, AII, BIII, and BIV. The distribution of the genotypes identified in the included Brazilian studies is presented in **Table 6**. Despite the limited number of molecular investigations, these studies provide important information on the circulation of *G. duodenalis* assemblages across different hosts and environmental matrices in Brazil.

**Table 6:** Distribution, frequency among studies, and epidemiological potential of the *Giardia duodenalis* assemblages identified in the Brazilian studies included in the systematic review (2016–2026).

Assemblage	Frequency (n)	Reported hosts/sources	Epidemiological potential	Included studies
A (AI/AII)	11	Humans, dogs, rabbits, pigs, goats, sheep, primates, bats, sloths, wild rodents and marsupials, water, and recreational environments	Zoonotic and anthroponotic; sub-assemblage AI predominant in wildlife; AII frequent in humans and production animals	Araújo et al. (2024); Baptista et al. (2023); Calegar et al. (2023); Corrêa et al. (2020); Fantinatti et al. (2016, 2020, 2023); Fehlberg et al. (2021); Harvey et al. (2023); Pineda et al. (2020); Reis et al. (2023); Reis et al. (2024); Seguí et al. (2018); Trevisan et al. (2020)
B (BIII/BIV)	6	Humans, non-human primates, bats, surface water, and reuse water	Predominantly anthroponotic, with zoonotic potential; high genetic diversity in Amazonian Indigenous populations	Araújo et al. (2024); Corrêa et al. (2020); Fantinatti et al. (2023); Harvey et al. (2023); Köster et al. (2021); Pereira Gonçalves et al. (2024); Reis et al. (2024)
C	2	Dogs	Specific to canids	Osmari et al. (2021); Trevisan et al. (2020)
D	1	Dogs	Specific to canids	Osmari et al. (2021)
E	4	Cattle, buffaloes, dogs, and humans (occasional detection)	Traditionally associated with production animals; recent reports in humans and dogs indicate potential zoonotic interface	Aquino et al. (2019); Fantinatti et al. (2016); Fantinatti et al. (2023); Harvey et al. (2023)

AI, AII, BIII, and BIV represent sub-assemblages that are molecularly distinct and frequently associated with different epidemiological profiles and host adaptation. A single study may report more than one assemblage; therefore, the total sum may exceed the number of molecular studies included.

Source: Authors.

In the studies conducted in humans, a predominance of assemblages A and B was observed, particularly of sub-assemblages AII, BIII, and BIV, frequently associated with anthroponotic transmission. Sub-assemblage AII was identified mainly in children and in collective environments, suggesting interpersonal transmission and fecal–oral contamination. Corrêa et al. (2020), in a public daycare center in São Paulo, concomitantly identified assemblages A and B in asymptomatic children, while Seguí et al. (2018) recorded a predominance of sub-assemblage AII in Paranaguá Bay, Paraná.

Assemblage B presented high genetic diversity, especially in Indigenous and Amazonian populations. Köster et al. (2021) observed a high frequency of this assemblage in Tapirapé Indigenous people in Mato Grosso, with wide intra-genotypic variability, suggesting intense local circulation of the parasite. In urban communities in Rio de Janeiro, Fantinatti et al. (2023) also identified marked occurrence of assemblage B in pediatric samples.

Although traditionally associated with production animals, assemblage E was occasionally detected in human samples, suggesting a potential zoonotic interface. Fantinatti et al. (2016, 2023) identified this genotype in children from urban communities and public daycare centers in Rio de Janeiro, while Harvey et al. (2023), in semi-rural communities of Ilhéus, Bahia, recorded the simultaneous occurrence of assemblages A, B, and E in children, indicating potential epidemiological sharing between humans and animals.

In domestic and production animals, a heterogeneous molecular distribution was observed. In dogs, assemblages C and D predominated, considered typical of canids, although assemblage A was occasionally identified, suggesting the potential participation of these animals in zoonotic cycles. Trevisan et al. (2020) identified a predominance of assemblage C in dogs from Cuiabá, Mato Grosso, with concomitant detection of assemblage A, while Osmari et al. (2021) recorded exclusively assemblages C and D in dogs from Rio Grande do Sul. In cattle and buffaloes, assemblage E predominated, as observed by Aquino et al. (2019) in buffalo calves. In pigs, goats, and sheep from rural communities in Piauí, Calegar et al. (2023) identified exclusively sub-assemblage AII, genetically similar to human isolates, suggesting potential interspecies transmission. In domestic rabbits, Baptista et al. (2023) detected assemblage A in predominantly asymptomatic animals, expanding the spectrum of hosts associated with zoonotically relevant giardiasis.

In wildlife, potentially zoonotic assemblages were identified in different hosts, including non-human primates, bats, rodents, marsupials, and sloths. Pereira Gonçalves et al. (2024) detected exclusively assemblage B in non-human primates in southern Brazil. Fehlberg et al. (2021) identified the AI subgenotype in wild rodents and marsupials from the Bahian Atlantic Forest, while Reis et al. (2023, 2024) recorded assemblages A and B in sloths and Amazonian bats, evidencing circulation of the parasite between natural and anthropized environments.

In environmental samples, sub-assemblages AII, BIII, and BIV were mainly identified. Araújo et al. (2024), in monitoring of surface and reuse water in the metropolitan region of São Paulo, characterized these genotypes, suggesting a predominantly anthropogenic origin of the environmental contamination. Pineda et al. (2020) confirmed assemblage A in samples from public swimming pools in Campinas, São Paulo, evidencing the potential for transmission in collective recreational environments.

Overall, the molecular findings demonstrate the circulation of both zoonotic and host-adapted assemblages across human, animal, and environmental compartments in Brazil, highlighting the epidemiological complexity of *Giardia duodenalis*. The predominance of assemblages A and B is consistent with their recognized epidemiological importance and broad host range. Nevertheless, the detection of identical assemblages in different hosts should be interpreted cautiously, as shared genotypes alone do not demonstrate direct zoonotic transmission but rather indicate potential epidemiological connectivity among human, animal, and environmental compartments (Ryan and Cacciò, 2013). Moreover, molecular characterization was performed in only a limited proportion of the included studies, and differences in genetic targets, amplification protocols, and genotyping strategies restrict direct comparisons among investigations. Consequently, although the occurrence of shared assemblages supports the existence of potential transmission interfaces, the available evidence remains insufficient to establish the direction or frequency of zoonotic transmission events. The assemblage distribution observed in Brazil is broadly consistent with that reported elsewhere. In Latin America, assemblages A and B likewise predominate, with sub-assemblages AII, BIII, and BIV most frequently identified (Sarria-Guzmán et al., 2022). In Africa, by contrast, assemblage B is markedly dominant, accounting for approximately 70% of genotyped cases, and assemblage E has not been detected in human samples (Walana et al., 2025). This contrast is epidemiologically relevant, since the occasional detection of assemblage E in Brazilian human and canine samples indicates a zoonotic interface that appears less evident in the African context. These findings reinforce the importance of expanding molecular surveillance using standardized methodologies integrated with epidemiological and environmental investigations to improve understanding of the transmission dynamics of giardiasis in Brazil within a One Health framework.

### 3.7 One Health implications of giardiasis in Brazil

The present systematic review provides the most comprehensive synthesis currently available on the occurrence of *Giardia* spp. in Brazil from a One Health perspective, integrating evidence from human, animal, and environmental studies published between 2016 and 2026. Collectively, the findings demonstrate that *Giardia duodenalis* circulates widely across multiple host species and environmental matrices, reinforcing the epidemiological complexity of giardiasis in the country. The detection of the parasite in humans, companion animals, livestock, wildlife, drinking water, sewage, food, recreational environments, and other environmental compartments indicates that transmission occurs through interconnected human–animal–environment interfaces rather than within isolated epidemiological cycles.

One of the principal findings of this review is that, despite the broad adoption of the One Health concept in recent years, genuinely integrated investigations remain uncommon in Brazil. Only nine of the 114 included studies simultaneously evaluated two or more epidemiological compartments, whereas most investigations focused exclusively on human, animal, or environmental populations. Consequently, although the available evidence consistently demonstrates the circulation of *Giardia* spp. across these compartments, the transmission interfaces connecting them remain insufficiently characterized. This fragmentation limits a comprehensive understanding of parasite transmission dynamics and highlights one of the main knowledge gaps identified in the Brazilian literature.

The simultaneous occurrence of *Giardia duodenalis* in humans, dogs, cats, ruminants, wild animals, and environmental matrices supports the occurrence of multiple transmission pathways in the country, involving human–animal–environment interfaces compatible with the One Health approach. Harvey et al. (2020, 2023), in semi-rural communities of Ilhéus, Bahia, demonstrated the concomitant circulation of the parasite in children, dogs, and environmental samples, with identification of assemblages A, B, and E in humans and predominance of assemblage E in canids, indicating a potential zoonotic interface. Toledo et al. (2017), in dairy farms in Paraná, recorded the simultaneous occurrence in cattle and in drinking water, while Breternitz et al. (2024) identified substantial positivity in bovine feces (46.1%) and in shallow wells used for human consumption (up to 47.8%), evidencing cross-contamination between herd and water sources.

The persistence of viable cysts in different environmental compartments reinforces the central role of the environment as an epidemiological link between humans and animals. Studies conducted in public supply systems demonstrated residual detection of *Giardia* spp. in treated water (Keller et al., 2024; Oliveira et al., 2024; Scherer et al., 2022; Silva et al., 2021), and Ladeia et al. (2022) and Martins F, et al. (2019) identified the maintenance of the parasite in effluents from sewage treatment plants, with viable forms in up to 30% of the final effluent. The molecular characterization by Araújo et al. (2024) identified human sub-assemblages (AII, BIII, and BIV) in surface and reuse water, suggesting a predominantly anthropogenic origin of the environmental contamination.

The distribution of studies reveals substantial regional heterogeneity, with greater concentration of research in the Southeast, Northeast, and South Regions of Brazil, while areas of the North and Central-West Regions remain relatively under-investigated. Despite this gap, studies conducted in the Brazilian Amazon and adjacent areas demonstrated high circulation of *Giardia duodenalis* in vulnerable populations and wildlife. Köster et al. (2021) recorded prevalences of 13.5% to 21.7% in Tapirapé Indigenous people, with high genetic diversity of assemblage B, while Zenazokenae et al. (2019) identified positivity of 9.3% in Haliti-Paresí Indigenous people in Mato Grosso. In wildlife, Reis et al. (2023, 2024) detected zoonotic assemblages in sloths and Amazonian bats, indicating that giardiasis also constitutes a relevant sanitary problem in areas of high socio-environmental vulnerability. These geographical disparities likely reflect differences in research capacity and surveillance infrastructure and emphasize the need to expand epidemiological investigations in historically underrepresented regions.

The epidemiological findings also reinforce the association between giardiasis and socioeconomic inequalities. Several studies identified higher prevalences in populations residing in peripheral areas, rural communities, Indigenous populations, Quilombola communities, and localities with deficient sanitary infrastructure. Eustachio et al. (2019), in a Quilombola community in Diamantina, Minas Gerais, recorded universal contamination of water samples by fecal coliforms; Capella et al. (2018) identified parasitic contamination in 100% of soil samples from a vulnerable community in Pelotas, Rio Grande do Sul; and Silva et al. (2025), in a rural community in Teresina, Piauí, demonstrated a significant association between human infection and contact with water bodies, inadequate waste disposal, and poor sanitation. These findings indicate a consistent association between parasitic infection, social vulnerability, and inadequate basic sanitation.

Although frequently underdiagnosed and neglected, giardiasis remains one of the most relevant enteric parasitic diseases in Brazil, particularly in children and populations exposed to inadequate hygiene and water supply conditions. The high frequencies observed in daycare centers and collective environments (Corrêa et al., 2020; Fantinatti et al., 2016, 2023), together with the detection of zoonotic assemblages in companion animals, production animals, and wildlife, support the interpretation that giardiasis should be addressed within an integrated human–animal–environment framework. In this context, the data compiled in this review reinforce the need for articulated strategies of epidemiological surveillance, environmental sanitation, veterinary control, and molecular monitoring, with a view to reducing the persistence and dissemination of the parasite across the different Brazilian ecosystems and to strengthening the national response to giardiasis from a One Health perspective.

### 3.8 Limitations of the review and future perspectives

This systematic review has some limitations that should be considered when interpreting its findings. First, substantial methodological heterogeneity was observed among the included studies, including differences in study design, sampling strategies, host populations, diagnostic techniques, molecular targets, and reporting formats. Such variability precluded quantitative synthesis through meta-analysis and limited direct comparisons of occurrence estimates across regions and host groups. In addition, the review protocol was not prospectively registered, and a formal risk of bias assessment was not performed because this review was designed to provide a descriptive synthesis of highly heterogeneous observational studies involving human, animal, and environmental compartments, with substantial variability in study designs and methodological approaches. The absence of prospective registration in PROSPERO or an equivalent platform is acknowledged as a specific limitation, distinct from the other methodological constraints, since pre-registration reduces the risk of reporting bias; this decision reflected the descriptive and exploratory scope of the review rather than a hypothesis-testing design. In addition, study selection and data extraction were performed by the principal author with verification by the co-authors, without the formal application of independent double screening, which is acknowledged as a methodological limitation. As independent double screening was not applied, no inter-rater agreement measure could be computed, and the possibility of selection or extraction errors cannot be fully excluded; verification by the co-authors was adopted to mitigate this risk. Consequently, the findings should be interpreted considering the methodological variability of the available evidence. Furthermore, molecular characterization was performed in only a small proportion of the included studies, restricting more robust inferences regarding the distribution of *Giardia duodenalis* assemblages and potential transmission interfaces.

The available evidence also reflects important gaps in the Brazilian literature. Most investigations evaluated human, animal, or environmental compartments independently, whereas only a limited number of studies simultaneously investigated these interfaces from a One Health perspective. Consequently, although the compiled evidence consistently demonstrates the widespread occurrence of *Giardia* spp. across different hosts and environmental matrices, it remains insufficient to establish transmission pathways or quantify the relative contribution of individual reservoirs to parasite circulation. These limitations should therefore be considered when interpreting the epidemiological relationships discussed throughout this review.

Future research should prioritize multidisciplinary studies integrating simultaneous human, animal, and environmental sampling, combined with standardized molecular characterization and harmonized diagnostic protocols. Expanding investigations in geographically underrepresented regions and socially vulnerable populations will also be essential to improve understanding of the epidemiology of giardiasis in Brazil. Such efforts will strengthen the evidence base required to support integrated surveillance and control strategies within a One Health framework.

## 4 Conclusion

The present systematic review demonstrates the broad distribution of *Giardia* spp. in Brazil, affecting humans, companion animals, production animals, wildlife, and multiple environmental matrices, highlighting the epidemiological complexity of *Giardia duodenalis* in the country. The compiled evidence indicates interconnected human, animal, and environmental compartments in parasite circulation, reinforcing the relevance of the One Health approach for understanding the epidemiology of giardiasis in Brazil.

Despite the broad body of evidence available, only nine of the 114 included studies (7.9%) simultaneously investigated human, animal, and environmental compartments, demonstrating that the One Health framing of the present review rests largely on the authors’ integrative synthesis rather than on the design of the underlying studies, and that important gaps remain in understanding transmission dynamics in Brazil. Expanding integrated One Health investigations should therefore be considered a priority for strengthening the epidemiological evidence available in the country.

The broad detection of assemblages A and B in humans, animals, and environmental samples, together with the occasional detection of assemblage E in humans and dogs, supports the occurrence of shared genotypes among different epidemiological compartments and suggests potential transmission interfaces. Likewise, the identification of the human-associated sub-assemblages AII, BIII, and BIV in surface and reuse water reinforces the contribution of environmental contamination to the maintenance and dissemination of giardiasis. However, because molecular characterization was available for only 17 of the 114 included studies (14.9%), the available molecular evidence remains insufficient to establish the direction or frequency of zoonotic transmission events, emphasizing the need for cautious interpretation of shared assemblages.

The reviewed studies also demonstrated a higher frequency of infection among socially vulnerable populations, particularly in settings characterized by inadequate sanitation, limited access to safe drinking water, and greater exposure to contaminated environments, reinforcing the importance of giardiasis as a persistent public health concern in Brazil.

Although advances have been achieved in diagnostic and molecular approaches, substantial methodological heterogeneity, the limited number of molecular investigations, the lack of standardized genotyping protocols, and the scarcity of integrated One Health studies continue to limit a more comprehensive understanding of transmission dynamics. Future multicenter studies integrating standardized molecular methods with simultaneous human, animal, and environmental investigations will be essential to strengthen epidemiological surveillance and support more effective prevention and control strategies for giardiasis in Brazil.

## Data Availability

The original contributions presented in the study are included in the article/supplementary material. Further inquiries can be directed to the corresponding author.
